# The Associations of Weekend Warrior Activity Patterns With the Visceral Adiposity Index in US Adults: Repeated Cross-sectional Study

**DOI:** 10.2196/41973

**Published:** 2023-01-11

**Authors:** Kai Wang, Fang Xia, Qingwen Li, Xin Luo, Jinyi Wu

**Affiliations:** 1 Department of Public Health Wuhan Fourth Hospital Wuhan China

**Keywords:** weekend warrior, Visceral Adiposity Index, NHANES, physical activity, obesity

## Abstract

**Background:**

According to previous reports, obesity especially visceral fat has become an important public health problem, causing an estimation of 20.5 disability-adjusted life years per 1000 inhabitants. Those who exercised for 1 or 2 days per week and reached the recommended 150 minutes of moderate physical activity (PA) per week have been defined as “weekend warriors” (WWs). Although the benefits of PA in suppressing obesity have been widely studied, the association of WWs with the Visceral Adiposity Index (VAI) and the difference between WW activity and regular PA are yet to be explored.

**Objective:**

This study aims to explore the association between WW activity and other PA patterns with VAI in US adults.

**Methods:**

The National Health and Nutrition Examination Survey 2007-2016 data set was used, and the analytic sample was limited to adults 20 years and older who had complete information about VAI, PA patterns, and other covariates, including demographic characteristics, behavioral factors, and disease conditions. Participants’ characteristics in different PA pattern groups were tested using the Rao and Scott adjusted *χ*^2^ test and ANOVA. Univariate and multivariate stepped linear regression models were then used to explore the association between the PA pattern and VAI. Finally, stratified analyses and interaction effects were conducted to investigate whether the association was stable among subgroups.

**Results:**

The final sample included 9642 adults 20 years or older, which is representative of 158.1 million noninstitutionalized US adults, with 52.15% (n=5169) being male and 70.8% (n=4443) being non-Hispanic White. Gender, age group, race, education level, income level, marital status, smoking status, alcoholism, VAI, cardiovascular disease, and diabetes were all correlated with the PA pattern, but no relationship between hypertension and PA pattern was observed. After adjusting for demographic covariates, smoking status, alcoholism, cardiovascular disease, diabetes, and hypertension, WW and regularly active adults had a β of .307 (95% CI –0.611 to –0.003) and .354 (95% CI –0.467 to –0.241), respectively, for reduced VAI when compared with inactive adults, but no significant effect of lowering VAI (β=–.132, 95% CI –0.282 to 0.018) was observed in insufficiently active adults when compared with inactive adults. Besides, no significant difference was exhibited between WW adults and regularly active adults (β=.047, 95% CI –0.258 to 0.352), suggesting WW adults had the same benefit of decreasing VAI as regularly active adults. Stratified analyses results exhibited that WW activity was related to reduced VAI in female adults aged 20-44 years who were non-Hispanic Black, other, or multiracial; high school or General Educational Development education; and never married, and the association between PA pattern and VAI remained stable in all demographic subgroups.

**Conclusions:**

Compared with inactive adults, WWs could reduce VAI, and there was no significant difference between WWs and regular active adults in decreasing VAI. Our study provides compelling evidence of the beneficial effect of WW activity on visceral obesity.

## Introduction

Physical activity (PA) has been proven to be beneficial by a number of studies, including for reducing chronic disease risk, improving mental state, and prolonging life [1-3]. The World Health Organization recommended that people aged 18 to 64 years should perform at least 150 minutes of moderate-intensity aerobic exercise per week, 75 minutes of high-intensity aerobic exercise per week, or an equivalent combination [4]. Randomized clinical trials have also shown that short-term periodic intermittent PA could improve cardiopulmonary health and other health outcomes, such as blood lipid levels and obesity [5].

In contrast to those who did 30 minutes of moderate-intensity PA for 5 days per week, those who did all exercise on 1 or 2 days per week were usually defined as “weekend warriors” (WWs) [6,7]. As society has become more fast paced worldwide, participating in sports frequently may be less suitable for a busy lifestyle, which has caused a gradual increase in the proportion of WWs. However, at present, the scientific community still knows little about WWs and whether their exercise schedule is more beneficial than inactivity or if WW activity had the same benefits as regularly active (RA) adults.

According to previous reports, obesity, especially visceral fat, has become an important public health problem, causing an estimation of 20.5 disability-adjusted life years per 1000 inhabitants. The economic impact of care for comorbidities associated with obesity could amount to US $2.158 billion. [8]. Obesity is related with various diseases, including cardiovascular disease (CVD), prodrome diabetes, type 2 diabetes mellitus (T2DM), hypertension, hyperlipidemia, sleep apnea, and some malignant tumors [9]. BMI as a routine weight measurement index has been widely used in scientific research [10,11]. However, BMI focuses on measuring overweight, which is not a reliable index to estimate body fat distribution. In contrast, the Visceral Adiposity Index (VAI) is a simple gender-specific indicator of visceral fat dysfunction, which can estimate body fat distribution. Moreover, VAI could be used as a risk predictor of functional impairment and disease, including for CVD, T2DM, nonalcoholic cirrhosis, or erectile dysfunction [12-18]. According to previous research, it is widely accepted that PA could reduce BMI and prevent obesity [10,19,20]. However, the association of WW activity with VAI and the difference between WW activity and regular PA remain to be explored.

Therefore, we obtained data from the National Health and Nutrition Examination Survey (NHANES) to conduct an analysis concerning WW activity and VAI. Through this research, we would like to make the public aware of the benefits of PA on VAI and make suggestions on how to reduce VAI to further reduce various metabolic disease risks. At the same time, the impact of different modes of PA on VAI is an interesting research direction. We also used large-scale data to analyze the different PA modes and VAI so as to provide guidance on the choice of PA mode.

## Methods

### Study Population

Data was derived from the six continuous NHANES cycles from 2007-2008 to 2017-2018, which is a nationally representative population-based survey for assessing adult and child health and nutritional status in the United States [21]. The examination components consisted of medical, dental, and physiological measurements, and laboratory tests, which were supervised by trained medical personnel. Furthermore, the adoption of various modern equipment and compensation for the participants enabled the NHANES to collect reliable and high-quality data.

**Figure 1:**
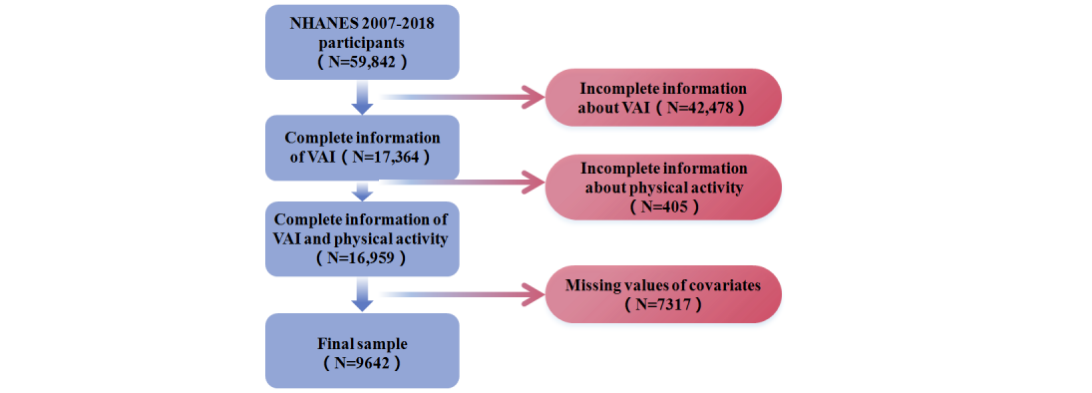
Flowchart of study design. NHANES: National Health and Nutrition Examination Survey; VAI: Visceral Adiposity Index.

The total sample size of adults from the 2007 to 2018 cycle was 9642. Additional details on the study design, sampling, and exclusion criteria are described in Figure 1.

### Ethics Approval

All participants in NHANES provide informed consent and NHANES is approved by the research ethics review board of the Centers for Disease Control and Prevention [22].

### Outcome Ascertainment

The VAI was measured with the following sex-specific formula: VAI = [waist circumference (WC) / 39.68 + (1.88 * BMI)] * (triglycerides / 1.03) * (1.31 / high-density lipoprotein cholesterol [HDL-C]) for males and VAI = [WC / 36.58 + (1.89 * BMI)] * (triglycerides / 0.81) * (1.52 / HDL-C) for females, in which triglycerides and HDL-C were expressed in mmol/L, WC expressed in cm, and BMI expressed in kg/m^2^ [23]. The Cobas 6000 Chemistry Analyzer is optimized for workloads using a combination of photometric and ion-selective electrode determinations (c501 module), and electrochemiluminescence technology in the detection of triglyceride concentration [24]. HDL-C was measured with a magnesium/dextran sulfate solution that was first added to the specimen to form water-soluble complexes with non–HDL-C fractions [25].

### PA and Other Covariates

PA in this study was assessed with a PA questionnaire in which participants were asked about the frequency and duration of vigorous and moderate sports, fitness, and recreational activities for at least 10 continuous minutes in a typical week. PA was calculated with the combination of frequency (times per week) and duration (duration per time), and total PA was calculated with the formula 2 * vigorous PA + moderate PA, since 1 minute of vigorous-intensity activity was defined as equivalent to 2 minutes of moderate-intensity activity according to PA guidelines [4]. Therefore, PA patterns were categorized into the following: inactive (no vigorous or moderate PA), insufficiently active (<150 minutes per week of total PA), WW (at least 150 minutes per week of total PA in 1 or 2 sessions), and RA (at least 150 minutes per week of total PA in more than 2 sessions).

Based on previous studies, covariates in this study included demographic data such as gender (male, female), age (20-44, 45-64, ≥65 years), race (non-Hispanic White, non-Hispanic Black, Mexican Americans, and other or multiracial races), education level (less than high school graduate, high school degree or equivalent, more than high school degree), income level (measured with as the ratio of family income to poverty [PIR]: low income PIR≤1.3, middle income 1.3<PIR<3.5, or high income PIR≥3.5), marital status (married or living with partner; divorced, separated, or widowed; never married). Behavioral risk factors included smoking status (never smoked, former smoker, current smoker) and alcoholism (yes, no). CVD was defined as a self-reported congestive heart failure, coronary heart disease, angina, myocardial infarction, or stroke diagnosed by a professional doctor. Diabetes was defined as a fasting plasma glucose ≥126 mg/dL, 2-hour plasma glucose ≥200 mg/dL, hemoglobin A_1c_ ≥6.5%, or self-reported diabetes diagnosed by a professional doctor. Hypertension was defined as an average systolic pressure ≥140 mm Hg and diastolic pressure ≥90 mm Hg in 3 tests [26-29].

### Statistical Analysis

According to analytic guidelines published by the National Center for Health Statistics, stratum and primary sampling units were taken into account for the complex, multistage, probability sampling design. Since 6 consecutive cycles were derived from NHANES, the 2-year mobile examination center weight divided by 6 was adopted to be representative of the general population.

Initially, participants’ characteristics in different groups were tested using the Rao and Scott [30] adjusted *χ*^2^ test and ANOVA. Univariate and multivariate binary stepped logistic regression models were then used to explore the association between PA pattern and depression risk. Model 1 was nonadjusted, while model 2 was adjusted for demographic data. Model 3 was adjusted for demographic data, behavioral factors, and disease condition. Finally, a stratified analyses and interaction effect were conducted to investigate whether the association was stable among subgroups. Statistical analyses were performed using the Stata software (version 16.0, StataCorp LLC). All statistical tests were 2-sided, and significance was considered at α=.05.

## Results

### Characteristics of Study Participants

As described in Figure 1, 16,059 participants with complete information about VAI and PA pattern were enrolled in the study, and 1542, 2417, 10, 2464, and 884 participants were excluded because of incomplete information about income level, marital status, smoking status, alcoholism, and hypertension, respectively. The final sample included 9642 adults 20 years and older, which is representative for 158.1 million noninstitutionalized US adults, with 52.15% (n=5169) being male and 70.8% (n=4443) being non-Hispanic White.

The characteristics of participants in different PA pattern groups are presented in Table 1. Gender, age group, race, education level, income level, marital status, smoking status, alcoholism, VAI, CVD, and diabetes were all correlated with the PA pattern, but no relationship between hypertension and the PA pattern was observed. To be more specific, WW adults were more likely to be male, be aged 20-44 years, be non-Hispanic White, have some college or above, have a PIR≥3.5, be married or living with a partner, have never smoked, have lower VAI, be nonalcoholic, have CVD, and have diabetes.

**Table 1:** Participants characteristics according to physical activity pattern.

Characteristics		Overall (n=9642)	Inactive (n=4854)	Insufficiently active (n=1474)	Weekend warrior (n=190)	Regularly active (n=3124)	*P* value	
**Gender, n (%)**								<.001
	Male	5169 (52.15)	2543 (50.70)	704 (47.96)	151 (73.45)	1771 (54.69)		
	Female	4473 (47.85)	2311 (49.30)	770 (52.04)	39 (26.55)	1353 (45.31)		
**Age group (years), n (%)**								<.001
	20-44	4074 (44.82)	1694 (38.09)	625 (44.00)	146 (51.70)	4609 (51.70)		
	45-64	3424 (37.78)	1877 (41.42)	536 (38.23)	40 (33.98)	971 (33.98)		
	≥65	2144 (17.40)	1283 (20.49)	313 (17.77)	4 (0.02)	544 (14.32)		
**Race, n (%)**								<.001
	Non-Hispanic White	4443 (70.80)	2192 (68.37)	704 (74.21)	56 (56.44)	1491 (73.00)		
	Non-Hispanic Black	1877 (9.43)	979 (10.53)	278 (8.30)	49 (14.54)	571 (8.34)		
	Mexican American	1398 (8.08)	786 (9.50)	180 (6.42)	38 (12.87)	394 (6.84)		
	Other or multiracial	1924 (11.69)	897 (11.61)	312 (11.07)	47 (16.16)	668 (11.83)		
**Education level, n (%)**								<.001
	Less than high school graduate	2119 (14.26)	1508 (22.15)	209 (9.41)	41 (17.34)	361 (6.65)		
	High school graduate	2226 (22.49)	1248 (27.14)	329 (21.90)	45 (19.08)	604 (17.26)		
	Some college or above	5297 (63.25)	2098 (50.71)	936 (68.69)	104 (63.58)	2159 (76.08)		
**Income level, n (%)**								<.001
	PIR^a^≤1.3	2916 (20.30)	1818 (27.11)	345 (15.78)	60 (23.01)	693 (13.88)		
	1.3<PIR<3.5	3660 (35.41)	1933 (38.94)	568 (35.15)	76 (40.50)	1083 (30.92)		
	PIR≥3.5	3066 (44.29)	1103 (33.95)	561 (49.07)	54 (36.48)	1348 (55.20)		
**Marital status, n (%)**								<.001
	Married or living with partner	5845 (64.72)	2949 (64.26)	912 (66.87)	104 (55.50)	1880 (64.80)		
	Divorced, separated, or widowed	2068 (17.80)	1201 (21.02)	312 (16.70)	23 (12.00)	532 (14.69)		
	Never married	1729 (17.48)	704 (14.73)	250 (16.43)	63 (32.51)	712 (20.51)		
**Smoking status, n (%)**								<.001
	Never	4800 (51.12)	2168 (45.36)	779 (53.79)	113 (63.09)	1740 (56.30)		
	Former	2649 (27.82)	1343 (27.18)	425 (29.20)	28 (14.75)	853 (28.67)		
	Current	2193 (21.07)	1343 (27.46)	270 (17.01)	49 (22.16)	531 (15.03)		
**Alcoholism, n (%)**								<.001
	No	7982 (83.85)	3858 (79.79)	1256 (85.23)	164 (87.88)	2704 (87.98)		
	Yes	1660 (16.15)	996 (20.21)	218 (14.77)	26 (12.12)	420 (12.02)		
**Cardiovascular disease, n (%)**								<.001
	No	8637 (91.85)	4199 (89.12)	1348 (93.72)	184 (98.70)	2906 (93.97)		
	Yes	1005 (8.15)	655 (10.88)	126 (6.28)	6 (1.30)	218 (6.03)		
**Diabetes, n (%)**								<.001
	No	7689 (85.04)	3615 (79.69)	1213 (87.48)	178 (94.75)	2683 (89.95)		
	Yes	1953 (14.96)	1239 (20.31)	261 (12.52)	12 (5.25)	441 (10.05)		
**Hypertension, n (%)**								.17
	No	9355 (97.44)	4697 (97.23)	1425 (96.72)	184 (98.12)	3049 (97.99)		
	Yes	287 (2.56)	157 (2.77)	49 (3.28)	6 (1.88)	75 (2.01)		
Visceral Adiposity Index, 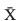 (SD)		1.98 (2.61)	2.24 (3.04)	1.95 (1.84)	1.71 (1.72)	1.68 (2.33)	<.001	

^a^PIR: ratio of family income to poverty.

### Relationship Between PA Pattern and VAI

**Figure 2:**
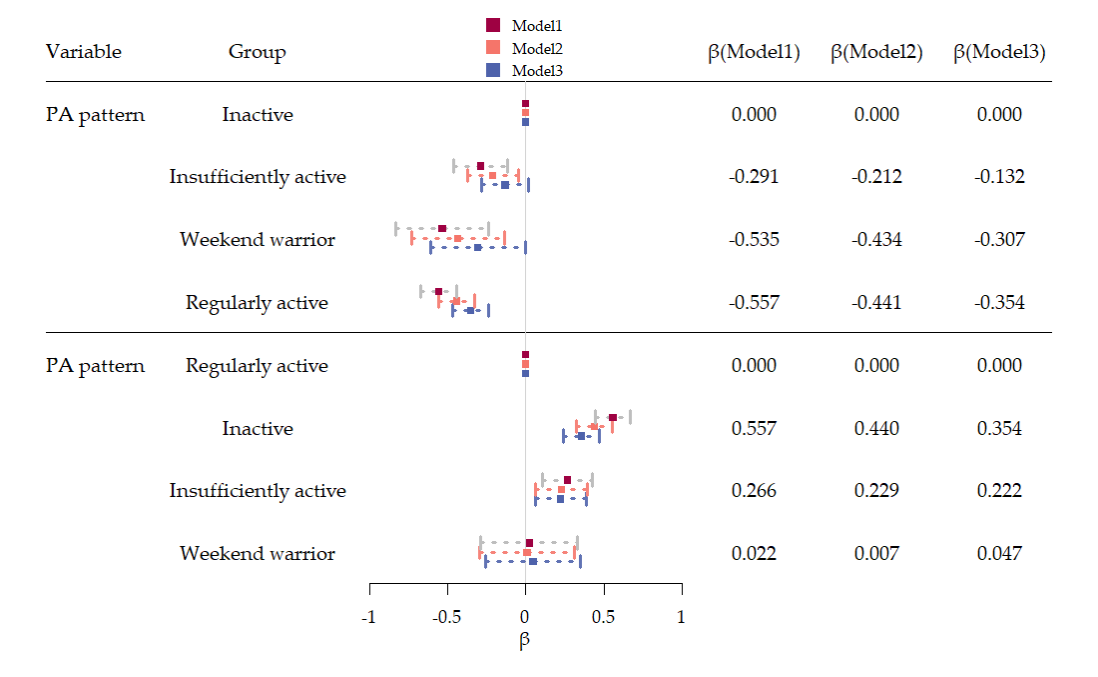
The forest plot of PA pattern and the Visceral Adiposity Index in adults. PA: physical activity.

The results of the binary univariate and multivariate logistic regression models of PA pattern on VAI were presented in Table 2 and Figure 2, and all models revealed a significantly lower VAI among WW and RA adults when compared with inactive adults. After adjusting for demographic covariates, smoking status, alcoholism, CVD, diabetes, and hypertension, WW and RA adults’ VAI reduced by 0.307 (95% CI –0.611 to –0.003) and 0.354 (95% CI –0.467 to –0.241), respectively, but no significant effect was observed for VAI reduction (β=–.132, 95% CI –0.282 to 0.018) in insufficiently active adults. Moreover, no significant difference was exhibited between WW adults and RA adults, suggesting WW adults had the same benefit of decreasing VAI as RA adults.

**Table 2:** The relationship between physical activity pattern and Visceral Adiposity Index in adults.

Characteristics	Model 1^a^		Model 2^b^		Model 3^c^			
	β (95% CI)	*P* value	β (95% CI)	*P* value	β (95% CI)	*P* value		
Inactive	Reference	N/A^d^	Reference	N/A	Reference	N/A		
Insufficiently active	–.291(–0.463 to –0.118)	.001	–.212 (–0.374 to –0.049)	.01	–.132 (–0.282 to 0.018)	.08		
Weekend warrior	–.535 (–0.830 to –0.239)	.001	–.434 (–0.732 to –0.135)	.005	–.307 (–0.611 to –0.003)	.04		
Regularly active	–.557 (–0.671 to –0.443)	<.001	–.441 (–0.556 to –0.326)	<.001	–.354 (–0.467 to –0.241)	<.001		
Regularly active	Reference	N/A	Reference	N/A	Reference	N/A		
Inactive	.557 (0.443 to 0.671)	<.001	.440 (0.326 to 0.556)	<.001	.354 (0.241 to 0.468)	<.001		
Insufficiently active	.266 (0.106 to 0.426)	.001	.229 (0.063 to 0.395)	.007	.222 (0.060 to 0.385)	.008		
Weekend warrior	.022 (–0.287 to 0.332)	.89	.007 (–0.294 to 0.309)	.96	.047 (–0.258 to 0.352)	.76		

^a^Model 1 was the univariate model in which no covariates were adjusted.

^b^Model 2 was adjusted for demographic covariates, including gender, age group, race, education level, income level, and marital status.

^c^Model 3 was additionally adjusted for smoking status, alcoholism, cardiovascular disease, diabetes, and hypertension.

^d^N/A: not applicable.

### Stratified Analyses According to Demographic Characteristics

To better understand the association between PA pattern and VAI in various subgroups, we conducted stratified analyses according to demographic characteristics. As described in Table 3, the WW activity pattern was related to a reduced VAI in female participants aged 20-44 years who were non-Hispanic Black, other, or multiracial, had a high school degree or General Educational Development, and were never married. The results of the interaction analyses revealed that all *P* values did not reach the significant level, indicating that the association between PA pattern and VAI remained stable in all demographic subgroups.

**Table 3:** The relationship between physically activity pattern and Visceral Adiposity Index in subgroups^a^.

Characteristics			Inactive		Insufficiently active, β (95% CI)		Weekend warrior, β (95% CI)	Regularly active, β (95% CI)		*P* value for interaction	
**Gender**											.17
	Male	Reference		–.123 (–0.363 to 0.118)		–.229 (–0.636 to 0.177)		–.373 (–0.547 to –0.199)			
	Female	Reference		–.152 (–0.334 to 0.030)		–0.621 (–0.902 to –0.339)		–.327 (–0.502 to –0.151)			
**Age group (years)**											.61
	20-44	Reference		–.262 (–0.483 to –0.041)		–.388 (–0.776 to 0.000)		–.425 (–0.603 to –0.247)			
	45-64	Reference		–.055 (–0.361 to 0.250)		–.314(–0.883 to 0.259)		–.269 (–0.558 to 0.021)			
	≥65	Reference		–.053 (–0.345 to 0.240)		–.251 (–0.608 to 0.107)		–.375 (–0.554 to –0.195)			
**Race**											.28
	Non-Hispanic White	Reference		–.148(–0.345 to 0.049)		–.270 (–0.744 to 0.204)		–.390 (–0.548 to –0.233)			
	Non-Hispanic Black	Reference		–.136 (–0.316 to 0.045)		–.345 (–0.600 to –0.089)		–.236 (–0.447 to –0.026)			
	Mexican American	Reference		–.414 (–0.744 to –0.083)		–.384 (–1.430 to 0.661)		–.198 (–0.578 to 0.183)			
	Other or multiracial	Reference		.093 (–0.243 to 0.429)		–.493 (–0.928 to –0.059)		–.370 (–0.615 to –0.125)			
**Education level**											.99
	Less than high school graduate	Reference		–.243 (–0.539 to 0.053)		–.154 (–0.722 to 0.414)		–.369 (–0.684 to –0.053)			
	High school graduate or GED^b^	Reference		–.241 (–0.533 to 0.052)		–.446 (–0.805 to –0.086)		–.361 (–0.596 to –0.125)			
	Some college or above	Reference		–.098 (–0.300 to 0.105)		–.271 (–0.727 to 0.184)		–.351 (–0.499 to –0.202)			
**Income level**											.58
	PIR^c^≤1.3	Reference		–.293 (–0.541 to –0.044)		–.225 (–0.660 to 0.211)		–.414 (–0.651 to –0.177)			
	1.3<PIR<3.5	Reference		–.111 (–0.388 to 0.166)		–.166 (–0.781 to 0.450)		–.220 (–0.474 to 0.034)			
	PIR≥3.5	Reference		–.108 (–0.323 to 0.107)		–.392 (–0.822 to 0.037)		–.429 (–0.587 to –0.272)			
**Marital status**											.16
	Married or living with partner	Reference		–.099 (–0.295 to 0.097)		–.188 (–0.722 to 0.347)		–.448 (–0.603 to –0.295)			
	Divorced, separated, or widowed	Reference		–.255 (–0.541 to 0.032)		–.287 (–0.767 to 0.194)		–.145 (–0.478 to 0.188)			
	Never married	Reference		–.121 (–0.474 to 0.231)		–.412 (–0.657 to –0.167)		–.168 (–0.437 to 0.101)			

^a^The models were adjusted for demographic characteristics, behavioral factors, and disease conditions.

^b^GED: General Educational Development.

^c^PIR: ratio of family income to poverty.

## Discussion

With NHANES, we found that the WW exercise pattern in US adults is helpful to reduce VAI compared with no exercise. In particular, there was no difference between WWs and RA adults, suggesting that WW activity had the same VAI reduction effect as regular activity. Moreover, stratified analysis and interaction analysis confirmed the stability of the results. This might suggest for people who cannot exercise regularly that WW activity could reduce visceral obesity and improve health.

To the best of our knowledge, this is the first analysis to explore the impact of PA patterns, especially of WWs, on VAI. At present, most studies focus on the influence of the sedentary behaviors and leisure time PA (LTPA) ratio on disease or health status [31,32]. Through a cohort study of a nationally representative sample of US cancer survivors, it was found that the combination of sedentary and physical inactivity was associated with the high risk of death [2]. Including the mode of PA adds to the analysis of the LTPA ratio and concerns not only the duration but also the frequency of exercise. Therefore, the NHANES database was used in this study to determine the effects of WW and other PA patterns on visceral fat distribution with VAI.

We took inactivity as the reference. In model 1, no covariates were considered, and insufficiently active, WW, and RA adults had reduced VAI. In model 2, after adjusting for demographic data, the results were similar to the univariate analysis. In model 3, WW and RA adults had reduced VAI after adjusting for demographic data, behavioral factors, and disease conditions, while no significant difference was observed in insufficiently active adults and inactive adults. In detail, RA adults had the largest negative correlation with VAI (β=–.35; *P*<.001), and WW adults also had an obvious negative correlation (β=–.31; *P*=.047). Eekelen et al [33] found that moderate-to-vigorous PA (MVPA) was associated with less body fat, visceral fat, and liver fat. Mild PA seems to be associated with less body fat but not visceral fat or liver fat. This suggests that exercise would preferentially reduce visceral fat compared to caloric restriction, which might be because visceral fat is more metabolically active and sensitive to lipolytic activation in the adrenal system. Similarly, inactive and insufficiently active adults had elevated VAI when compared with RA adults. Nevertheless, no significant difference was observed in WW and RA adults, indicating the importance of PA duration rather than frequency.

Moreover, the linear regression showed other influencing factors of VAI (see Multimedia Appendix 1 for details). Using no exercise or regular exercise as a reference, age, race, income, marriage, smoking, and diabetes all had an impact on VAI. In the age group, there was no difference in VAI between those aged 20-44 years and those aged 45-64 years, but adults 65 years or older were significantly negatively correlated with VAI, which might be caused by emaciation in older adults. Cameron et al [11] reported the interaction between age and PA. They found that a decrease in BMI and body fat percentage was greater in the older adult group than in the younger group, which was consistent with our results.

Our results showed that non-Hispanic Black participants were more likely to reduce VAI than non-Hispanic White participants. Some studies found that, after correcting for total fat mass, Black women had less visceral adipose tissue than White women, while White people had more visceral adipose tissue than African American people [10]. African American people were more likely to reduce visceral adipose tissue, which supported our results.

There was no difference between middle-income and low-income groups on VAI, while the VAI of the high-income group was significantly lower than that of the low-income group. Ameye and Swinnen [34] reported that obesity varies with income but in a nonlinear way. Overall, obesity in low-income countries increased with greater income but was not related with income in middle-income countries, while it decreases with greater income in high-income countries. Since our data set was from the United States, it was in line with Ameye and Swinnen [34], and it may be related to social stigma around overweight and good medical resources in high-income countries.

Unmarried people were more likely to reduce VAI than married people. According to Lee et al [35], the prevalence of abdominal obesity in married participants was higher than those with other marriage statuses, which was consistent with our results. This marriage-related difference might be due to the fact that marriage increased the frequency of meals and snacks, thus increasing total energy consumption. Compared with married people, unmarried people were more eager to lose weight and maintain weight to make themselves more attractive for future marriage.

Smokers might have higher VAI than nonsmokers. Wehby et al [36] used a sample of 1057 mothers from Norway and found a heterogeneous effect of smoking on BMI. With an increase in smoking, BMI would increase at low/medium BMI levels, while BMI would decrease at high BMI levels. They observed genetic effects on the relationship between smoking and increased BMI, and gene analysis (eg, *CHRNA3*) had consistent evidence that increased BMI was related with smoking, which may explain our results.

Participants who were diabetic could increase VAI more than those without diabetes. According to Haslam’s [37] review, experimental results showed that after 6 months of overfeeding, the BMI of young men without a family history of diabetes would increase to 28.0 kg/m^2^, and the levels of fasting plasma insulin, glucose, and triglyceride would also increase reversibly, thereby impairing glucose tolerance. About 90% of patients with type 2 diabetes had a BMI greater than 23.0 kg/m^2^. Patients who are diabetic might take drugs that cause weight gain, while vulnerable individuals who are already obese may take drugs that cause hypoglycemia.

VAI has been proven by many studies to be a predictor of cardiovascular and metabolic diseases such as hypertension, T2DM, CVD, and nonalcoholic fatty liver disease. Therefore, we planned to conduct a structural equation model in the next study to analyze the mediation effect of VAI: whether the activity pattern might affect VAI and related diseases. This future research direction would improve the relationship between exercise, diet, and visceral obesity, and further specify the analysis of activity and diet patterns.

There were several advantages in this study. First, we adopted a large-scale data set including 9642 participants, thus ensuring representativeness. Second, the topic of activity and obesity was specified in this study, thus the association between visceral obesity and different exercise modes. Finally, this study used a hierarchical linear regression method to analyze the covariates of VAI in detail. However, there were still some limitations. First, this study was based on a cross-sectional investigation, which could not infer the causal relationship between activity patterns and VAI. Second, there are many factors related to VAI, and the variables included in this study are limited. Third, since this study focused on the activity patterns, it did not further explore the relationship between exercise intensity, exercise/sedentary ratio, and VAI, which could be explored in the next study.

In conclusion, although the benefits of physical activities were well known, many people had limited time to engage in activities, and MVPA only accounted for 5% of the total time for daytime activities. Therefore, the role of WW activity is worth researching. According to our results, we found that, compared with no activity, WWs could reduce VAI, and there was no difference between WWs and RA adults, indicating that WW activity is worth promoting for people who cannot exercise regularly. This research also suggested that the duration of PA was more important than the frequency of activity. With this study, we want to make the public aware of the benefits about WW PA and encourage those who are tired from work but want to keep healthy to exercise during the weekend to reduce VAI.

## Appendix

Multimedia Appendix 1 Other risk factors of Visceral Adiposity Index.

## Abbreviations

CVD: cardiovascular disease
HDL-C: high-density lipoprotein cholesterol
LTPA: leisure time physical activity
MVPA: moderate-to-vigorous physical activity
NHANES: National Health and Nutrition Examination Survey
PA: physical activity
PIR: ratio of family income to poverty
RA: regularly active
T2DM: type 2 diabetes mellitus
VAI: Visceral Adiposity Index
WC: waist circumference
WW: weekend warrior

## Data Availability

The data sets were derived from NHANES 2007-2008 to 2017-2018 [38], and only publicly available data was used in this study. The data sets generated or analyzed during this study are available from the corresponding author on reasonable request.
