# Supplementary material for: The Associations of Weekend Warrior Activity Patterns With the Visceral Adiposity Index in US Adults: Repeated Cross-sectional Study
Source: JMIR Public Health Surveill. 2023 Jan 11;9:e41973. doi: 10.2196/41973 (PMC9878365; doi:10.2196/41973)
Supplement: Multimedia Appendix 1 [file publichealth_v9i1e41973_app1.docx]

Supplemental Table 1 Other risk factors of VAI

| **Variables** | **Features** | **OR (95% CI)** | **P value** |
| --- | --- | --- | --- |
| **Gender** | Male | ref |  |
|  | Female | -0.061(-0.185,0.063) | .33 |
| **Age group** | 20-44 years | ref |  |
|  | 45–64 years | 0.079(-0.079,0.237) | .32 |
|  | ≥65 years | -0.454(-0.674,-0.234) | <.001 |
| **Race** | Non-Hispanic White | ref |  |
|  | Non-Hispanic Black | -0.783(-0.934,-0.632) | <.001 |
|  | Mexican American | 0.144(-0.079,0.368) | .20 |
|  | Other or multiracial | -0.093(-0.266,0.080) | .29 |
| **Education level** | Less than high school graduate | ref |  |
|  | High school graduate | -0.088(-0.360,0.183) | .52 |
|  | Some college or above | -0.045(-0.305,0.215) | .73 |
| **Income level** | PIR ≤ 1.3 | ref |  |
|  | 1.3 < PIR < 3.5 | -0.008(-0.164,0.147) | .92 |
|  | PIR ≥ 3.5 | -0.255(-0.400,-0.109) | <.001 |
| **Marital Status** | Married or living with partner | ref |  |
|  | Divorced, separated, or widowed | 0.131(-0.052,0.314) | .16 |
|  | Never married | -0.201(-0.361,-0.041) | .01 |
| **Smoking status** | Never | ref |  |
|  | Former | 0.115(-0.018,0.248) | .09 |
|  | Current | 0.278(0.107,0.449) | .002 |
| **Alcoholism** | No | ref |  |
|  | Yes | -0.043(-0.241,0.154) | .66 |
| **CVD** | No | ref |  |
|  | Yes | 0.302(-0.0378,0.643) | .08 |
| **Diabetes** | No | ref |  |
|  | Yes | 1.125(0.840,1.410) | <.001 |
| **Hypertension** | No | ref |  |
|  | Yes | 0.293(-0.061,0.647) | .10 |

PIR, ratio of family income to poverty; CVD, cardiovascular disease.
